# Visual straight-ahead preference in saccadic eye movements

**DOI:** 10.1038/srep23124

**Published:** 2016-03-15

**Authors:** Damien Camors, Yves Trotter, Pierre Pouget, Sophie Gilardeau, Jean-Baptiste Durand

**Affiliations:** 1 Université de Toulouse, Centre de Recherche Cerveau et Cognition, Toulouse, France; 2 Centre National de la Recherche Scientifique, Toulouse Cedex, France; 3 Inserm U975, Movement disorders and basal ganglia, Hôpital de la Salpêtrière, Paris, France; 4 Université Pierre & Marie Curie-Paris 6, CNRS UMR 7225, CR-ICM, UMR S975, Paris, France; 5 Institut de Neurosciences Translationnelles de Paris, IHU-A-ICM, Paris, France

## Abstract

Ocular saccades bringing the gaze toward the straight-ahead direction (centripetal) exhibit higher dynamics than those steering the gaze away (centrifugal). This is generally explained by oculomotor determinants: centripetal saccades are more efficient because they pull the eyes back toward their primary orbital position. However, visual determinants might also be invoked: elements located straight-ahead trigger saccades more efficiently because they receive a privileged visual processing. Here, we addressed this issue by using both pro- and anti-saccade tasks in order to dissociate the centripetal/centrifugal directions of the saccades, from the straight-ahead/eccentric locations of the visual elements triggering those saccades. Twenty participants underwent alternating blocks of pro- and anti-saccades during which eye movements were recorded binocularly at 1 kHz. The results confirm that centripetal saccades are always executed faster than centrifugal ones, irrespective of whether the visual elements have straight-ahead or eccentric locations. However, by contrast, saccades triggered by elements located straight-ahead are consistently initiated more rapidly than those evoked by eccentric elements, irrespective of their centripetal or centrifugal direction. Importantly, this double dissociation reveals that the higher dynamics of centripetal pro-saccades stem from both oculomotor and visual determinants, which act respectively on the execution and initiation of ocular saccades.

There is a wide consensus regarding the fact that ocular saccades bringing the eyes toward their central orbital position (centripetal saccades) have higher dynamics than those steering the eyes away from the center (centrifugal saccades). Notably, centripetal saccades have been repeatedly described as being executed faster than centrifugal saccades of matched amplitude, with shorter durations and higher peak velocities^1^,^2^,^3^,^4^,^5^,^6^. Converging evidences also suggest that centripetal saccades are initiated more rapidly than centrifugal saccades following the appearance of visual elements^7^,^8^,^9^,^10^,^11^,^12^,^13^.

Oculomotor determinants have been put forward to explain the higher dynamics of centripetal saccades. Those “re-centering” saccades might face lower mechanical constraints^14^ and be driven by more efficient neural command signals^6^, explaining why they are executed faster. They might also benefit from enhanced preparatory activity in the superior colliculus^9^,^12^ and in other cortical structures involved in the regulation of saccades^10^, leading to more rapid initiations. However, centripetal and centrifugal saccades also differ in that they target visual elements lying closer and farther away from the straight-ahead direction respectively. This point is worth considering because elements appearing straight-ahead have already been shown to trigger manual responses more rapidly than elements in eccentric locations^17^. In the primary visual (V1) area, responses evoked by straight-ahead elements are also stronger than those elicited by eccentric elements, even if they form identical images on the retinas. This finding, first established at the neuronal level in macaques^15^,^16^, and further confirmed by functional imaging in humans^18^, indicate that the visual processing of the elements we are facing is prioritized. As a consequence of this privileged visual processing, elements located straight-ahead might thus trigger ocular saccades more efficiently than elements in more eccentric locations, just as it seems to elicit more efficient manual responses.

The aim of the present study is to assess the potential involvement of this visual determinant in the higher dynamics of “re-centering” saccades. In order to dissociate the centripetal or centrifugal direction of the saccades from the straight-ahead or eccentric location of the visual elements triggering those saccades, we have introduced anti-saccade tasks, in which participants perform saccades not toward but away visual elements. Classical pro-saccade tasks are expected to produce results in agreement with the literature, *i.e.* faster execution and more rapid initiation for centripetal saccades toward elements located straight-ahead than for centrifugal saccades toward eccentric elements. Anti-saccade tasks should thus disentangle whether this higher dynamic is maintained for centripetal saccades, despite the eccentric location of the triggering visual elements or for saccades evoked by straight-ahead elements, despite their centrifugal direction.

## Methods

### Participants

Twenty participants (10 females and 10 males; mean age ± standard deviation = 28.1 ± 3.8 years) were involved in the experiment. All of them had normal vision or corrected to normal with contact lenses or glasses, and reported no history of neurological or psychiatric disorders. They provided written informed consent before the experiment, which met the requirement of the ethical principles of the declaration of Helsinki and was approved by the local ethic committee (CLERIT). The experiment was carried out in accordance with the approved guidelines.

### Apparatus

**Figure 1:**
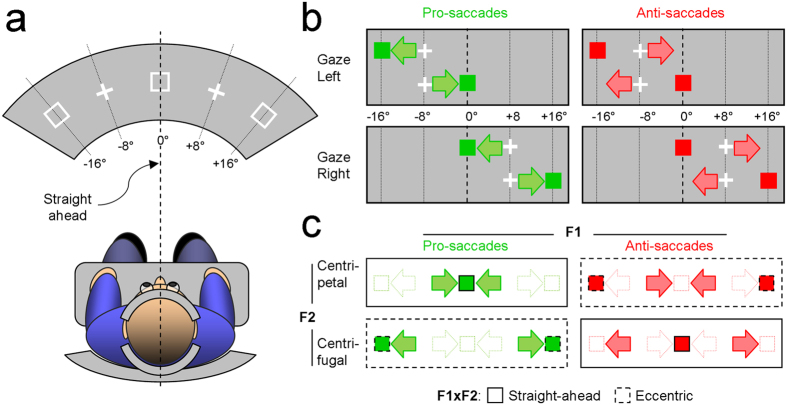
(**a**) Schematic representation of the experimental set-up. **(b)** The 4 types of block, that differed in the type of saccades (pro-saccades/anti-saccades) and in the initial gaze direction (8° left/8° right). Crosses are the initial fixation targets. Square symbols indicate the visual elements triggering saccades, which are symbolized by the arrows. **(c)** Factorial design produced by grouping the experimental conditions according to the types of saccades (F1: pro-saccades/anti-saccades) and to the directions of saccades (F2: centripetal/centrifugal). The interaction of these factors specifies the location of the visual elements (F1 × F2: straight-ahead/eccentric).

Participants sat in a chair, legs uncrossed, hands on a table. They positioned their heads within a head-support device clamped on top of the table and equipped with both chin and forehead supports. The uprights were further covered with sheets of dense foam in order minimize the possibility of head rotation during the experiment. The chair and head-support device were positioned so as to ensure a fine alignment between the participants’ head and trunk axes (Fig. 1a). Participants were asked to keep this positioning as constant as possible during the whole experiment. The experimenter checked the postural alignment of the subject after every block, and corrections were made when necessary. Participants faced a large and curved screen, subtending 180° × 45° at a viewing distance of 162 cm. Stimuli were displayed by a video projector (NEC NP1250) set to run with 70 Hz refresh rate at 800 × 600 pixels resolution. The experiment was controlled by the Psychophysics Toolbox extensions version 3.0^19^,^20^ installed on Matlab^®^ R2011 software, running on an Intel Core i5 based computer. A video-based binocular eye tracker (Eye Link 1000 desktop) placed 35 cm in front of the participants was used to record the movements of both the left and right eyes at 1 kHz per eye during the experiment.

### Experimental design

The participants alternated between 4 types of blocks (Fig. 1b), which differed with respect to the type of saccades: pro- (left column) or anti- (right column) saccades, and with respect to the initial gaze direction: 8° left (upper row) or right (lower row) relative to the straight-ahead direction. In total, each participant performed 12 blocks of 50 trials (3 repetitions of each type of block) in a single session of about 1 hour. At the beginning of each trial, participants maintained their gaze at the center (±1.5°) of a white fixation cross (0.5° × 0.5°) during a random period of 0.5 to 1 s. Successful fixations led to the appearance of a red or green square (1° × 1°), pseudo-randomly positioned 8° left or right from the fixation cross. In the blocks of pro-saccades, the participants had to move their gaze toward that visual element and to maintain fixation on its center (±1.5°) for 0.5 s. In the blocks of anti-saccades, the participants moved their gaze away and kept fixation on the location opposite to that of the visual element (±1.5°). In both cases, participants were instructed to respond as fast as possible. They were familiarized with both tasks by a training of 5 trials per task. Participants were instructed before each block regarding the task (pro- or anti-saccades), which was further signaled during the blocks by the color of the visual elements (green for pro-saccades and red for anti-saccades). The calibration of the eye tracker was adjusted every 4 blocks. As shown in Fig. 1c, the experimental conditions were grouped according to a 2 by 2 factorial design with the types (pro-saccades/anti-saccades; F1) and the directions (centripetal/centrifugal; F2) of the saccades as main factors. The interaction term specifies the locations of the saccades-triggering visual elements (straight-ahead/eccentric; F1 × F2). Note that by measuring both the left and right eyes and by balancing the leftward and rightward saccades in all the experimental conditions shown in Fig. 1c, we insured that the laterality of the saccades could not act as a confounding factor on our results and conclusions.

### Data analysis

**Figure 2:**
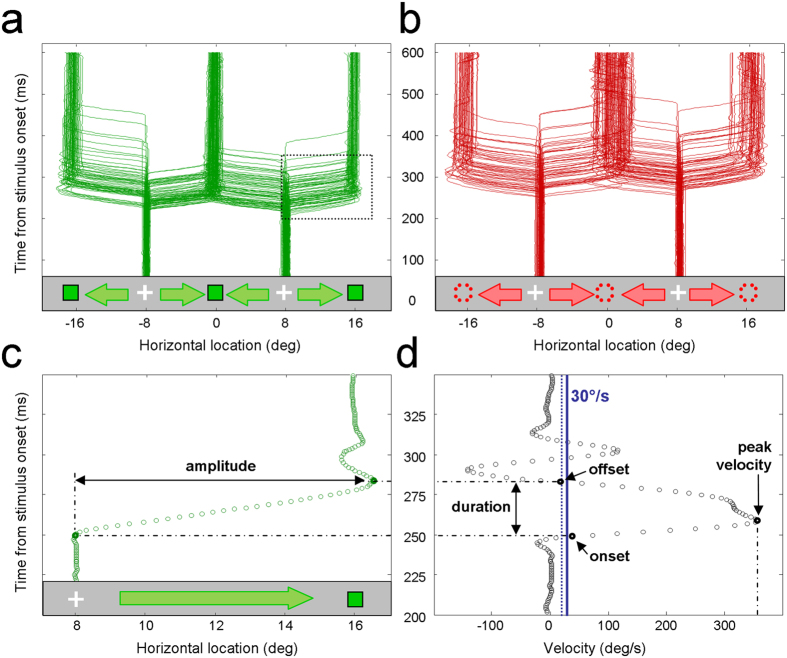
(**a**) Binocular traces for pro-saccades collected on an exemplar participant (average of horizontal locations for the left and right eyes as a function of post-stimulus time, in milliseconds). **(b)** Binocular traces for anti-saccades collected on the same subject. **(c)** Exemplar pro-saccade trace within the spatio-temporal window shown in Fig. 2a (dotted rectangle). **(d)** Corresponding velocity profile used to determine the onset, offset, duration and peak velocity of the saccade (see Methods). The amplitude of the saccade is determined as the angular horizontal deviation in eyes position between the onset and offset of the saccade (Fig. 2c). The blue vertical line indicates the velocity threshold used to detect the onset and offset of the saccade (30°/sec). Note that lowering the threshold to 20°/s (blue dotted line) would change neither the onset nor the offset in this particular example.

The positions of the left and right eyes, sampled at 1 kHz per eye, were averaged to produce binocular traces which were analyzed offline. Trials were discarded if: (1) the traces were incomplete around the times of the saccades (3.9% of the total number of trials, 468/12000 trials), (2) the first saccade was in the wrong direction (2.6%), (3) the amplitude of the first saccade was less than 6° (5.1%) or more than 10° (1.4%), so that the gaze reached the desired location through a second corrective saccade, or, (4) the saccade onset revealed an anticipation (<100 ms; 1.1%) or an attentional lapse (>800 ms; 2.3%). For the good trials (83.6% of the total number of trials, 10032/12000), the traces were differentiated in order to get velocity profiles. Traces collected from an examplar subject are provided in Fig. 2a for pro-saccades and Fig. 2b for anti-saccades. Figure 2c,d focus on an individual trace around the time of the saccade and its corresponding velocity profile.

As shown in Fig. 2d, the onsets, offsets and durations of the saccades were extracted from these velocity profiles as the beginning, the end and the length of the periods during which the velocity exceeded 30°/s. Two additional parameters were extracted: (1) the peak velocities, *i.e.* the maximum velocities reached by the saccades (Fig. 2d) and (2) the amplitudes of saccades, *i.e.* the differences in horizontal eyes positions between the onsets and offsets of saccades (Fig. 2c). As illustrated in Fig. 2d, lowering the velocity threshold from 30°/s (solid blue line) to 20° (dashed blue line) had no, or negligible, influence on those different parameters. Overall, our conclusions were found to be unaffected by the choice of a particular velocity threshold between 20 and 30°/sec. We characterized the dynamics of execution of the saccades by their peak velocities (in degrees per second). The dynamics of initiation were apprehended through the promptness of the saccades (in second^−1^), which correspond to the inverse of the onsets (also named latencies or reaction times). We favored the use of promptness values, rather than onsets, because they are normally distributed and are positively related to the speed of initiation (while onsets have right-skewed distributions, negatively related to the speed of initiation). For each subject and each of the 4 experimental conditions detailed in Fig. 1c, the mean peak velocity, mean promptness values and mean amplitudes were computed, with their 95% confidence intervals obtained through bootstrapping. These parameters were analyzed following the 2 by 2 factorial design illustrated in Fig. 1c, through 2-way repeated measures ANOVA. Within-subject differences in onsets, durations and offsets were also computed separately for the pro- and anti-saccades and these differences were submitted to Student’s *t*-tests. The significance threshold was set at 1%. All the analyses were performed with Matlab^®^ and its Statistical toolbox^®^.

## Results

### Oculomotor determinants in the execution of centripetal versus centrifugal saccades

**Figure 3:**
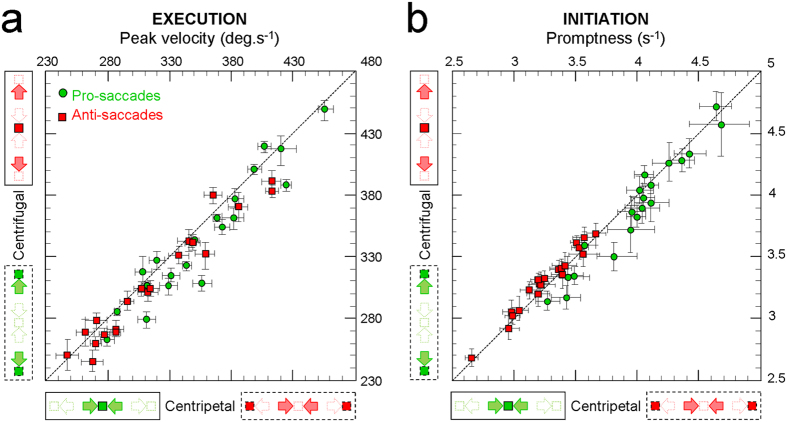
(**a**) Dynamics of saccades execution. Individual mean peak velocities (and 95% confidence interval) of centripetal versus centrifugal saccades, both for the pro-saccades (green circular symbols) and for the anti-saccades (red square symbols). **(b)** Dynamics of saccades initiation. Individual mean promptness (and 95% confidence interval) of centripetal versus centrifugal saccades. Same conventions as **(a)**. Results and statistics at the level of each participant are provided as Supplementary material.

Are centripetal pro-saccades executed faster than centrifugal pro-saccades because they bring the eyes toward their primary (central) position or because they are triggered by visual elements located straight-ahead? We addressed this issue by considering the peak velocities of centripetal and centrifugal pro- and anti-saccades. The mean peak velocities (and 95% confidence interval) for each of the 20 participants are shown in Fig. 3a, both for the pro-saccades (green circular symbols) and for the anti-saccades (red square symbols).

This figure calls for 2 main observations. First, the distribution of peak velocities for the pro-saccades tends to lie above that for the anti-saccades along the identity diagonal (the black dashed diagonal), reflecting higher peak velocities for pro-saccades (mean ± standard deviation: 351.0 ± 49.3 deg/s) than for anti-saccades (313.7 ± 48.4 deg/s). Second, for both types of saccades, the distributions are located mostly below the identity diagonal. This point is important because it reveals that centripetal saccades are executed faster than centrifugal saccade for both pro-saccades, triggered by straight-ahead elements (centripetal: 357.1 ± 48.5 deg/s; centrifugal: 344.9 ± 51.2 deg/s), and anti-saccades, triggered by eccentric elements (centripetal: 318.2 ± 50.4 deg/s; centrifugal: 309.2 ± 47.0 deg/s).

The statistical significance of these observations was assessed by the 2-way repeated measure ANOVA with the types of saccades (pro-saccades/anti-saccades) and the directions of saccades (centripetal/centrifugal) as main factors and the location of the visual elements (straight-ahead/eccentric) as the interaction term (see Methods). The results, shown in Table 1, confirm that both the types and directions of saccades have a highly significant impact on their peak velocities, while the locations of the visual elements show no influence. It can be concluded that centripetal saccades are executed faster than centrifugal saccades, irrespective of whether they are triggered by visual elements located straight-ahead or in eccentric locations.

### Visual determinants in the initiation of centripetal versus centrifugal saccades

As for the execution of saccades, we asked whether the more rapid initiation of centripetal pro-saccades is caused by the re-centering direction of those saccades or by the fact that they are elicited by visual elements located straight-ahead. The question was addressed by considering the promptness of centripetal and centrifugal pro- and anti-saccades saccades, shown in Fig. 3b with similar conventions as those used in Fig. 3a.

Once again, the distribution for the pro-saccades lies above that for the anti-saccades along the identity diagonal, indicating that pro-saccades are initiated more promptly (3.93 ± 0.41 s^−1^) than anti-saccades (3.28 ± 0.26 s^−1^). This is in agreement with previous reports^21^,^22^. For the pro-saccades, the distribution is mostly located below the identity diagonal, with a mean promptness of 3.98 ± 0.39 s^−1^ for centripetal pro-saccades, against 3.89 ± 0.44 s^−1^ for centrifugal pro-saccades. In striking contrast, most data points for the anti-saccades lie above the identity diagonal, with a larger mean promptness for centrifugal anti-saccades (3.30 ± 0.25 s^−1^) than for centripetal anti-saccades (3.26 ± 0.26 s^−1^). Although those promptness differences between centripetal and centrifugal saccades are statistically significant when considering separately the pro-saccades (Student’s t-test; t = 3.84, p = 0.001) and the anti-saccades (t = −3.45, p = 0.003), they cancel out when grouping those 2 types of saccade (t = 1.67, p = 0.108). This indicates that the directions of saccades *per se* cannot explain the observed differences.

Importantly, these observations were confirmed by the 2-way repeated measure ANOVA since the promptness of saccades was found to be very significantly impacted by the types of saccades (pro-saccades/anti-saccades) but not by their directions (centripetal/centrifugal). The significance of the interaction term reveals that the dynamics of initiation of the saccades depend on the location of the visual elements triggering those saccades (straight-ahead/eccentric). Together, these results show that saccades are initiated faster when they are triggered by visual elements located straight-ahead, irrespective of whether they bring the eyes toward (centripetal) or away (centrifugal) the center of the orbits.

### Functional consequences on the time courses of centripetal and centrifugal saccades

**Figure 4:**
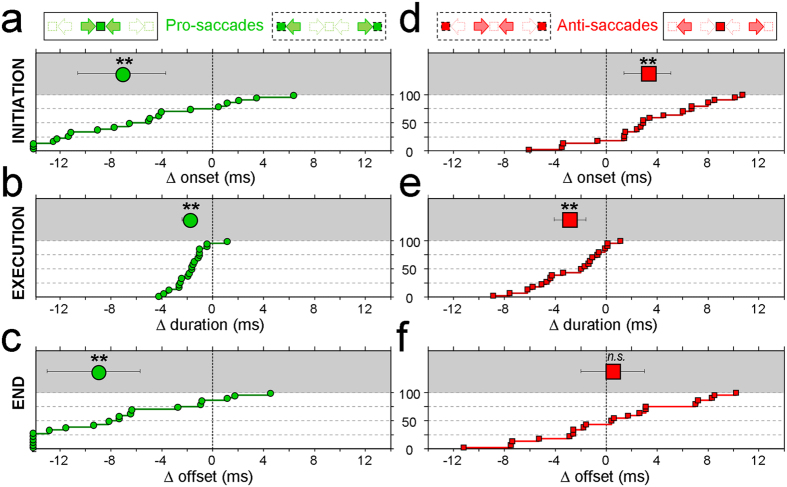
(**a–c**) Differences in the dynamics of centripetal and centrifugal pro-saccades, for the onsets (**a**), durations (**b**) and offsets (**c**) of the saccadic eye movements. Individual differences are sorted in the bottom part of each plot (white background) and the mean differences (with 95% confidence intervals) are shown on top (grey background). **(d–f)** Differences in the dynamics of centripetal and centrifugal anti-saccades. Data points showing differences greater than ±14 ms are stacked along the corresponding vertical axes. The asterisks indicate statistically significant (p < 10^−2^) deviations of the distributions means from 0, as assessed by Student’s t-tests (*n.s.*: non-significant).

We have found that centripetal pro-saccades are initiated more rapidly because they are triggered by visual elements located straight-ahead (visual determinants), and executed faster because they bring the eyes toward their primary position (oculomotor determinants). Thus, both mechanisms converge for bringing the eyes earlier on visual elements located straight-ahead than on eccentric locations. This was assessed by considering the differences in onsets, durations and offsets between centripetal and centrifugal pro-saccades. The results are shown in Fig. 4a–c for the individual participants (bottom part of each plot; white background) and for the group (mean and 95% confidence interval in the upper part; grey background). On average, centripetal pro-saccades are initiated 7.2 ms (95% CI: 3.4–10.8 ms) more rapidly (Fig. 4a) and they last 1.8 ms (1.3–2.4 ms) less than centrifugal pro-saccades of matched amplitude and directions. As a result, centripetal pro-saccades reach their desired locations 9.1 ms (5.6–12.9 ms) earlier than centrifugal saccades (Fig. 4c). Student’s *t*-tests indicate that all these differences are statistically highly significant (Δ onsets: t = 3.91, p < 10^−3^; Δ durations: t = 6.52, p < 10^−5^; Δ offsets: t = 4.81, p < 10^−3^).

We have shown the same determinants to be at work for anti-saccades. However, they have opposite effects, since centripetal anti-saccades are executed faster because they re-center the eyes in their orbits, but are initiated more slowly because they are triggered by visual elements occupying eccentric locations. These subtractive actions are shown in Fig. 4d–f. Centripetal anti-saccades are initiated 3.4 ms (1.3–5.2 ms) more slowly than centrifugal pro-saccades (Fig. 4d), but they are executed 2.9 ms (1.7–4.1 ms) faster (Fig. 4e). As a net result, centripetal and centrifugal anti-saccades tend to reach their desired locations at about the same time, with a slight advantage of 0.6 ms (−2.0–3.1) for the centrifugal anti-saccades (Fig. 4f). Once again, Student’s *t*-tests confirm that the differences in onsets and durations are very significant (Δ onsets: t = 3.32, p < 10^−2^; Δ durations: t = 4.46, p < 10^−3^), but they cancel each other in the case of anti-saccades (Δ offsets: t = 0.45, p = 0.66).

### Amplitude of centripetal and centrifugal saccades

**Figure 5:**
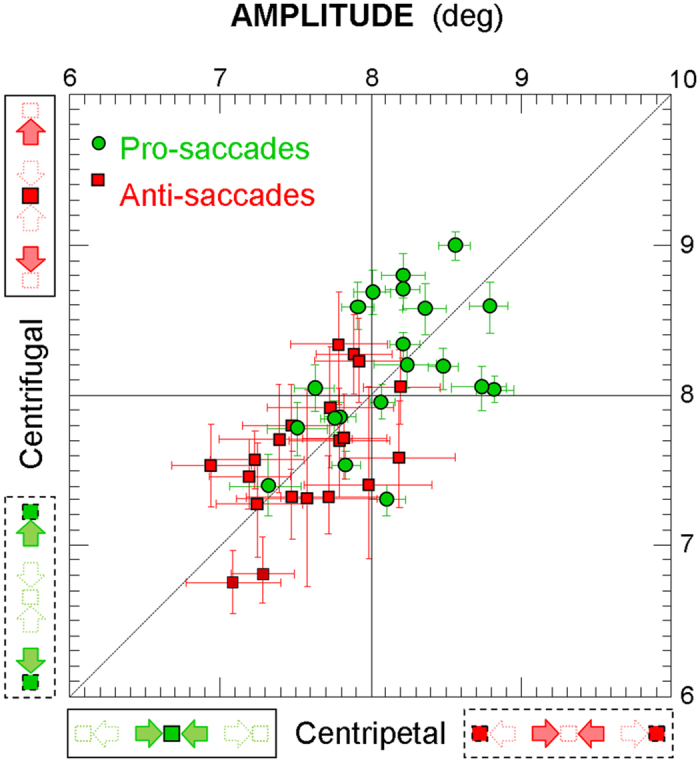
Individual mean saccade amplitude (and 95% confidence interval) of centripetal versus centrifugal saccades, both for the pro-saccades (green circular symbols) and for the anti-saccades (red square symbols).

Are the differences in dynamics between centripetal and centrifugal pro- and anti-saccades somehow related to variations in the amplitude of those saccades? Figure 5 shows the individual mean saccade amplitude (and 95% confidence interval) of centripetal versus centrifugal saccades, for both pro-saccades (green circular symbols) and anti-saccades (red square symbols).

The mean amplitude (±standard deviation) of pro-saccades (8.15° ± 0.39°) is close to the expected amplitude (8°) and significantly larger than that of anti-saccades (7.31° ± 0.36°; 2-way repeated measure ANOVA: F = 19.7, p < 10^−3^ for the saccade type). However, for both pro- and anti-saccades, data points are distributed evenly above and below the identity diagonal, indicating that the amplitude of saccades is consistently influenced neither by their direction (F = 0.3, p = 0.60) nor by the location of the saccade-triggering elements (F = 0.0, p = 0.94). For the anti-saccades, the absence of visual landmarks on the expected landing positions also induces a larger dispersion of landing eye positions (standard deviation = 0.94° ± 0.14°) than that observed for pro-saccades (0.52° ± 0.13°; F = 291.7, p < 10^−12^). This highly significant difference can be apprehended by comparing the length of the confidence interval bars between anti-saccades (in red) and pro-saccades (in green) in Fig. 5. However, once again, no difference is found between centripetal and centrifugal saccades (F = 1.4, p = 0.25), nor between saccades triggered by elements located straight-ahead or eccentric (F = 0.4, p = 0.54). Together, these results indicate that both the higher peak velocity of centripetal saccades and the shorter latency of saccades triggered by straight-ahead elements are unrelated to variations in the amplitude or precision of those saccadic eye movements.

### Reproducibility test of the saccadic onset results

**Figure 6:**
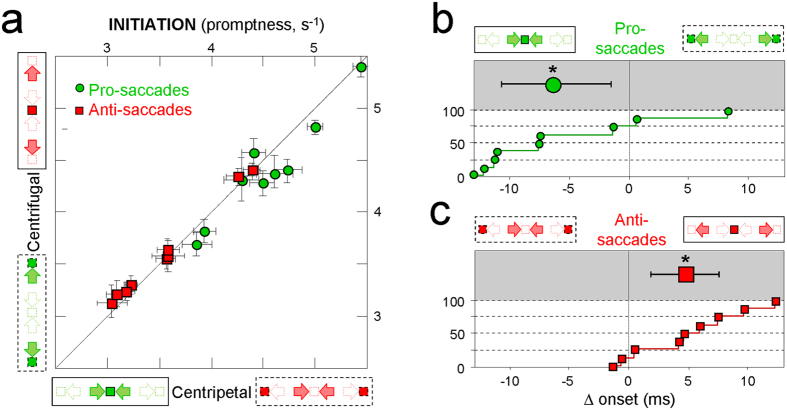
(**a**) Individual mean promptness (and 95% confidence interval) of centripetal versus centrifugal saccades, both for the pro-saccades (green circular symbols) and for the anti-saccades (red square symbols). **(b,c)** Differences in the onset of centripetal and centrifugal pro-saccades **(b)** and anti-saccades **(c)**. The asterisks indicate statistically significant (p < 0.05) deviations of the distributions means from 0, as assessed by Student’s t-tests.

The experiment performed by 3 of the co-authors (D.C., Y.T. and J-B.D.) with 20 subjects at the Purpan hospital of Toulouse was also carried out by the other 2 co-authors (P.P. and S.G.) on 9 distinct subjects at the Salpêtrière hospital of Paris. Although the experimental design was similar to that shown in Fig. 1b,c, slight differences in the recordings and in the task instructions prevented mixing the two data sets. Those differences concerned (1) the eye tracking measures: monocular at 500 Hz, against binocular at 1 kHz in the main experiment and (2) the blocks of anti-saccades: no constraint was provided regarding the amplitude of the anti-saccades, while they were required to land at 8° (±1.5°) in the main experiment. This secondary data set was nevertheless used as a reproducibility test for the main result of the present study, *i.e.* the shorter initiation of saccades triggered by straight-ahead elements, irrespective of their centripetal or centrifugal directions. As shown in Fig. 6a, the results of this reproducibility test were qualitatively similar to those shown in Fig. 3a, with higher promptness values for pro-saccades than for anti-saccades and higher promptness values for saccades triggered by straight-ahead elements (centripetal pro-saccades and centrifugal anti-saccades). This was confirmed statistically by a 2-way repeated measure ANOVA, indicating a highly significant contribution of the saccades types (F = 91.2, p < 10^−4^), no contribution of the saccades directions (F = 1.4, p = 0.26) but, crucially, a significant interaction between those main factors (F = 15.7, p < 5.10^−3^). The shorter onsets of both centripetal pro-saccades (median: −6.4 ms 95%CI: [−10.7–−1.7] ms; Student’s t-test: t = −2.59, p = 0.03; see Fig. 6b) and centrifugal anti-saccades (median: 4.8 ms 95%CI: [2.1–7.5] ms; Student’s t-test: t = 3.17, p = 0.01; see Fig. 6c) are in the ranges of those found in the main experiment (see Fig. 4a,d).

## Discussion

Centripetal pro-saccades have been consistently shown to exhibit higher dynamics than centrifugal pro-saccades, with both faster executions and more rapid initiations. So far, these temporal advantages had been attributed solely to oculomotor determinants: centripetal saccades are more efficient because they pull the eyes back toward their primary orbital position. In the present study, we investigated whether the higher dynamics of centripetal saccades might stem from the fact that they are usually triggered by visual elements lying closer to the straight-ahead direction. We dissociated the centripetal/centrifugal directions of the saccades from the straight-ahead/eccentric locations of the visual elements triggering those saccades by contrasting pro- and anti-saccade tasks. Our results clearly indicate that centripetal saccades are always executed faster than centrifugal saccades (higher peak velocities and shorter durations), irrespective of whether the visual elements triggering those saccades are located straight-ahead (for pro-saccades) or in eccentric positions (for anti-saccades). These findings are in line with those of previous studies^1^,^2^,^3^,^4^,^5^,^6^ and they support the view that the faster execution of centripetal saccades has oculomotor determinants; i.e. lower mechanical constraints and/or greater efficiency of the command signals^6^,^14^. The absence of visual determinant is also supported by the observation that memory-triggered saccades (not triggered by visual elements), also exhibit a centripetal over centrifugal advantage in peak velocity^23^.

A different pattern of results emerged when considering the initiation of saccades. In agreement with previous reports^7^,^8^,^9^,^10^,^11^,^12^,^13^, we show that centripetal pro-saccades are initiated more rapidly than centrifugal pro-saccades. The difference found here (about 7 ms) is similar to that reported by Krebs and colleagues^9^,^10^ with pro-saccades of similar amplitude. Importantly, anti-saccades reveal an opposite pattern of results: centrifugal anti-saccades exhibit greater promptness than the centripetal ones. To our knowledge, this finding represents the first ever reported instance of greater dynamics for saccades bringing the eyes away from their primary position. These results indicate that saccades triggered by visual elements located straight-ahead are initiated earlier than those elicited by eccentric elements, irrespective of whether they steer the eyes closer or farther away from their central orbital position. Thus far, the enhanced preparatory activity observed in subcortical^9^,^12^ and cortical^10^ structures for centripetal pro-saccades had been interpreted as a saccadic “re-centering” bias. Taken together, our results show that this bias is rather a visual “straight-ahead” bias, echoing the recent finding that visual elements located straight-ahead receive a privileged visual processing in the early visual cortex^15^,^16^,^18^. This privileged visual processing might be inherited by the structures supporting the regulation of saccades through cortico-cortical and cortico-tectal connections, where it would translate into enhanced preparatory activity. A similar mechanism is likely to operate for other types of visually-driven actions, since manual responses evoked by visual elements located straight-ahead have already been shown to be more rapid than those evoked by elements in eccentric locations^17^.

Mechanistically, both oculomotor and visual determinants shape the differences between the dynamics of centripetal and centrifugal saccades. Those determinants artificially cancel each other in the case of anti-saccades, but ecologically act in concert to bring the eyes nearly 10 ms earlier on visual elements located straight-ahead than on elements in more eccentric locations. In the present study, the saccades had fixed desired amplitude of 8°. This amplitude was chosen because it is about midway in the range of saccades usually performed by humans^24^ and, considering the initial eye positions (±8°), those gaze shifts do not usually trigger head movements in natural, head-free conditions^25^. Whether additional mechanisms come into play for larger saccades, in which both head and eye movements are involved in shifting the gaze^8^ will remain to be addressed.

To conclude, the present work brings evidences that the straight-ahead direction is a cardinal direction for both the sensory and motor aspects of vision. It has been shown that the gaze is preferentially brought along that direction when starting the exploration of visual scenes^26^. Even when the gaze is shifted away, visual elements along that particular direction receive a privileged processing in peripheral vision^15^. As shown in the present study, this privileged processing contributes in bringing the eyes back toward that direction more promptly. Thus, the present findings highlight the tight interplay between the sensory and motor aspects of vision for insuring a special monitoring of the visual elements we are facing. This special monitoring is likely to reflect the ecological importance of the elements lying straight-ahead, either as potential obstacles during navigation or as optimal targets for visually-guided actions.

## Additional Information

**How to cite this article**: Camors, D. *et al.* Visual straight-ahead preference in saccadic eye movements. *Sci. Rep.* **6**, 23124; doi: 10.1038/srep23124 (2016).

## Supplementary Material

Supplementary Information

## Figures and Tables

**Table 1:** Results of the 2-way repeated measures ANOVA for the peak velocity (execution) and promptness (initiation) variables.

	EXECUTION			INITIATION		
	Peak velocity (deg/s)			Promptness (s^−1^)		
	mean Δ (95% CI)	F Value	p value	mean Δ (95% CI)	F value	P value
**F1: Types of saccades**(pro-saccades - anti-saccades)	**37.5(29.4–45.2)**	**42.6**	**<10**^**−5**^	**0.65(0.56–0.76)**	**103.9**	**<10**^**−8**^
**F2: Directions of saccades**(centripetal - centrifugal)	**10.6(6.3–14.9)**	**15.1**	**<10**^**−3**^	0.03(−0.01–0.06)	2.7	0.11
**F1xF2: Locations of visual elements**(straight-ahead - eccentric)	1.7(−3.8–7.4)	1.3	0.27	**0.07(0.04–0.10)**	**31.6**	**<10**^**−4**^

For both variables, the first column indicates the mean difference (and 95% confidence interval) across participants. The second and third column provides the related F and p values. Statistically significant results (p<0.01) are reported in bold and underlined.
