# Supplementary material for: Visual straight-ahead preference in saccadic eye movements
Source: Sci Rep. 2016 Mar 15;6:23124. doi: 10.1038/srep23124 (PMC4792160; doi:10.1038/srep23124)
Supplement: Supplementary Information [file srep23124-s1.pdf]

Supplementary Material

Visual straight-ahead preference in saccadic eye movements

Damien Camors[1,2], Yves Trotter[1,2], Pierre Pouget[3,4], Sophie Gilardeau[5], and Jean-Baptiste Durand[1,2*]

[1] Université de Toulouse, Centre de Recherche Cerveau et Cognition, Toulouse, France

[2] Centre National de la Recherche Scientifique, Toulouse Cedex, France

[3] Inserm U975, Movement disorders and basal ganglia, Hôpital de la Salpêtrière, Paris, France

[4] Université Pierre & Marie Curie-Paris 6, CNRS UMR 7225, CR-ICM, UMR S975, Paris, France

[5] Institut de Neurosciences Translationnelles de Paris, IHU-A-ICM, Paris, France

* Corresponding author: jbdurand@cerco.ups-tlse.fr.

Supplementary Text. Individual results

Supplementary Figure 1. Differences between centripetal and centrifugal saccades

Supplementary Table 1. Individual factorial analyses (2-way ANOVA)

Supplementary Text

Supplementary Figure 1a shows that most participants (15/20) exhibit higher peak velocity for centripetal pro-saccades (positive Δ, in green) and centripetal anti-saccades (positive Δ, in red). Only 3 participants (s17, s18, s19) have a reversed pattern and 2 participants (s8, s20) show mixed results. On average, the peak velocity of centripetal saccades is higher than that of centrifugal saccades, by ~12°/sec for pro-saccades and by ~9°/sec for anti-saccades (**Supplementary Figure 1b**).

Supplementary Figure 1c indicates that most participants also show greater promptness for centripetal pro-saccade (15/20; positive Δ, in green) and for centrifugal anti-saccades (16/20; negative Δ, in red). Although these differences are significant at the group level (**Supplementary Figure 1d**), most of them fail to reach significance at the level of the individual participants (i.e. confidence intervals crossing the baseline). This is due to the fact that the latency of saccades is notoriously variable, so that the differences in promptness values (mean absolute difference = 0.07 s^{-1}), are about an order of magnitude smaller than their inherent variability (mean standard deviation = 0.53 s^{-1}).

Supplementary Table 1 (left part) shows that peak velocities are significantly higher ($p<0.05$) for pro-saccades *versus* anti-saccades (F1) in all participants, for centripetal versus centrifugal saccades (F2) in 12 participants, and for straight-ahead versus eccentric elements (F1xF2) in only 1 participant. **Supplementary Table 1 (right part)** shows that promptness is significantly higher for pro-saccades *versus* anti-saccades (F1) in all participants, for centripetal versus centrifugal saccades (F2) in only 2 participants, and for straight-ahead versus eccentric elements (F1xF2) in 9 of them.

Overall, the pattern of results evidenced at the group level is largely confirmed at the level of the individual participants.

Supplementary Figure 1. (a) Individual differences in the execution of centripetal *versus* centrifugal pro-saccades (green) and anti-saccades (red). (b) Related group averages. (c) Individual differences in the initiation of centripetal *versus* centrifugal saccades. (d) Related group averages. Error bars indicate the 95% confidence intervals. Participants are sorted as a function of their Δ peak velocity for pro-saccades.

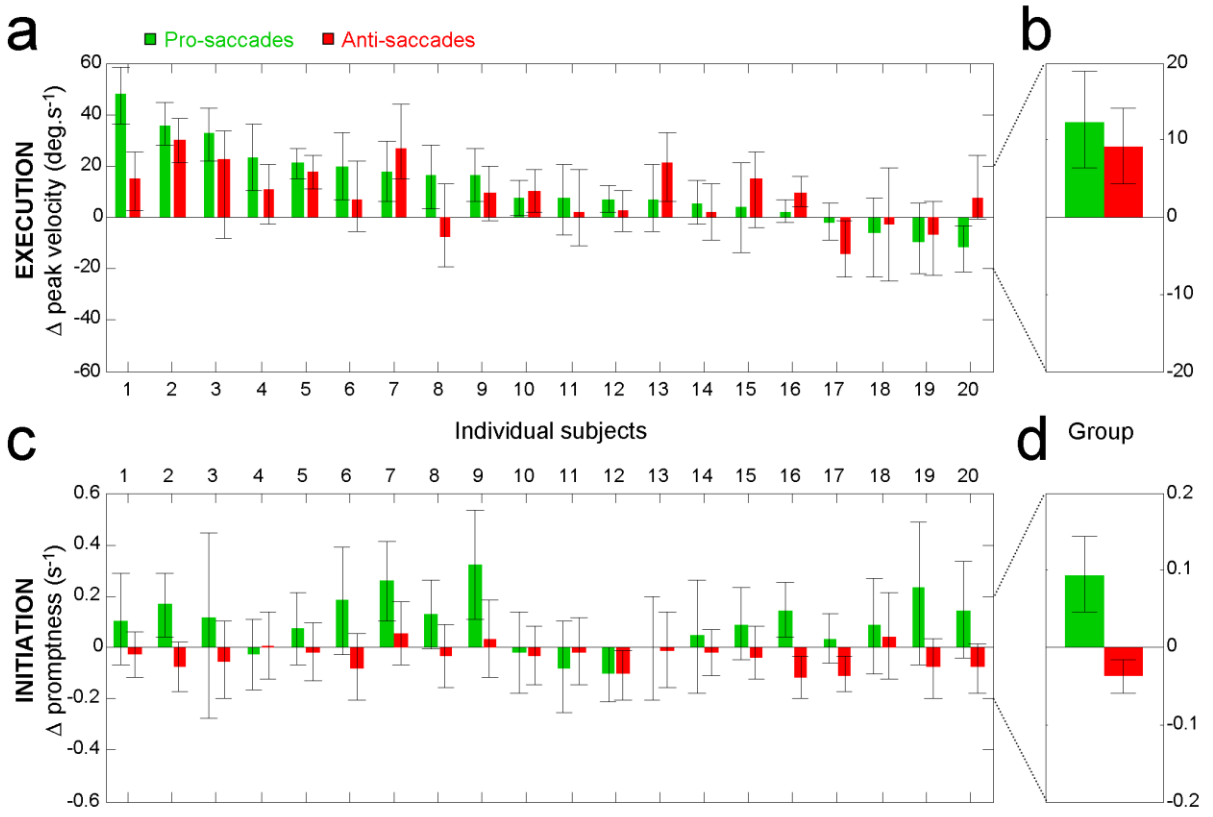

Supplementary Table 1. Mean individual differences in execution (peak velocity) and initiation (promptness) of saccades as a function of the types of saccades (F1: pro-saccades versus anti-saccades), their directions (F2: centripetal versus centrifugal) and the locations of the saccade-triggering visual elements (F1xF2: straight-ahead versus eccentric). Significant differences (2-way ANOVA; $p<0.05$) are underlined and indicated in red (positive) and blue (negative).

	**EXECUTION**			**INITIATION**		
	∆ Peak velocity (deg/s-1)			∆ Promptness (s-1)		
	**F1**	**F2**	**F1xF2**	**F1**	**F2**	**F1xF2**
**s01**	52,97	30,78	18,28	0,90	0,04	0,08
**s02**	8,36	33,34	4,89	0,66	0,06	0,13
**s03**	31,20	24,24	13,15	1,37	0,04	0,07
**s04**	13,27	17,09	9,23	0,39	-0,01	-0,02
**s05**	55,36	20,09	-2,24	0,62	0,04	0,04
**s06**	35,89	14,11	7,32	0,41	0,06	0,14
**s07**	17,07	23,05	-4,31	0,35	0,16	0,11
**s08**	43,39	8,70	8,82	0,17	0,06	0,08
**s09**	8,02	13,42	4,33	0,29	0,18	0,15
**s10**	42,79	8,41	-0,59	0,48	-0,03	0,00
**s11**	59,27	4,38	2,86	0,93	-0,11	0,01
**s12**	34,83	6,18	1,78	1,00	-0,03	-0,05
**s13**	51,90	13,87	-5,52	0,83	0,00	0,00
**s14**	49,90	3,04	3,04	0,69	-0,01	0,04
**s15**	40,45	4,97	0,12	0,94	0,01	0,11
**s16**	14,60	6,02	-3,76	0,17	0,02	0,13
**s17**	27,26	-5,96	4,03	0,53	-0,02	0,05
**s18**	72,62	-3,45	-4,84	0,83	0,09	0,02
**s19**	48,50	-6,94	0,07	0,82	0,09	0,16
**s20**	66,05	-0,89	-10,98	0,69	0,05	0,13
